# Ageism in HIV Research

**DOI:** 10.1093/geroni/igaa057.3106

**Published:** 2020-12-16

**Authors:** Tonya Taylor

**Affiliations:** SUNY Downstate Health Sciences University, Brooklyn, New York, United States

## Abstract

Sex and sexuality are important determinants of health and wellbeing across the life course. The desire and capacity for sexual intimacy and pleasure among older adults are neglected areas of research due to ageist assumptions that they no longer engage in sexual activity. These assumptions are most pronounced in HIV research, where we aggressively studied intimate details of sexual behaviors of people living with HIV until they became “old.” Interest in the sexual behaviors among older adults with HIV has waned in HIV prevention, suggesting an inherent ageism within the field. We will discuss emerging new HIV and STI risks for older adults, declining trends in gerosexuality funding, HIV media campaigns targeted for older adults, and new evidence that suggest that interventions that engage older adults with HIV in conversations about sexual health, menopause, and erectile dysfunction may be an effective strategy for promoting overall successful aging

